# A systematic review on patient perceptions and clinician‐reported outcomes when comparing digital and analog workflows for complete dentures

**DOI:** 10.1111/jopr.13999

**Published:** 2024-12-29

**Authors:** Amira Fouda, James Tonogai, Peter McDermott, Daniel Wang, Cecilia S. Dong

**Affiliations:** ^1^ Graduate Prosthodontic Resident, Faculty of Dentistry University of British Columbia Vancouver Canada; ^2^ Private Practice Hamilton Canada; ^3^ Schulich School of Medicine and Dentistry Department of Dentistry Western University London Canada; ^4^ Schulich School of Medicine and Dentistry Department of Pathology and Laboratory Medicine Western University London Canada

**Keywords:** clinician‐reported outcomes, complete, conventional, denture, digital, patient satisfaction

## Abstract

**Purpose:**

To compare digitally fabricated complete dentures to conventionally fabricated dentures using patient‐ and clinician‐reported outcome measures.

**Methods:**

This review was structured according to PRISMA guidelines with the protocol registered in the PROSPERO database (CRD42024526069). An electronic search of the databases with a defined search strategy was completed within PubMed/MEDLINE and Web of Science from January 2000 to March 2024. Grey literature and article references were searched. Articles were screened by title and abstract, and the remaining articles were screened by full‐text review. Articles accepted for inclusion were subjected to a risk‐of‐bias assessment using Cochrane Collaboration tools (RoB 2 and ROBINS‐I).

**Results:**

From an initial pool of 704 articles, 15 studies met the selection criteria, of which the majority were published within the past 3 years. Within the included studies, there was inconsistency in the assessment methods of patient‐ and clinician‐reported outcomes, making it challenging to draw definitive conclusions. Generally, digital dentures had superior cost‐effectiveness and prosthesis fabrication time. Patient satisfaction and denture quality were not consistently improved with digital technology.

**Conclusions:**

Studies showed indications of patient satisfaction with digital and conventional dentures. Digital technology may enhance clinical workflows. A trend emerged that milled dentures performed better than printed dentures. Clinicians adopting digital technology into removable prosthodontics may have a learning curve to overcome, and they should consider the patient‐clinician relationship in addition to clinical outcomes to achieve patient satisfaction. Additional studies with standardized tools for assessing patient satisfaction are required to enable meaningful comparisons between digital and conventional workflows.

Complete denture therapy is an important treatment option for the completely edentulous population^1^ and fabrication of complete dentures has traditionally relied on the manual skills and artistry of clinicians and dental technicians. With the advent of computer‐aided design and computer‐aided manufacturing (CAD‐CAM), the skillset, workflows, and fabrication techniques for complete dentures have undergone a shift in focus.^2^, ^3^

Digital technology is transforming the traditional workflow for removable prosthodontics.^4^, ^5^ Although digital technology was initially introduced in the discipline of fixed prosthodontics^6^, ^7^, digital concepts in complete denture therapy have advanced significantly since the first article on the digital fabrication of a set of complete dentures was published by Maeda et al. in 1994.^8^ After Maeda's landmark article, various researchers and clinicians have explored the concepts of CAD‐CAM fabricated complete dentures. Research in this field has often focused on denture‐related outcomes such as fit and accuracy; however, there may be gaps in the literature regarding clinician preferences for the digital workflow and its integration into daily practice. In addition, understanding the differences between digital and conventional workflows in terms of patient satisfaction, esthetics, comfort, retention, and trust in the treatment process may be beneficial. Clinicians today practicing removable prosthodontics should be aware of topics such as intraoral scanning,^9^ digital design processes, ^10^, ^11^, ^12^, ^13^ and additive/subtractive lab workflows.^14^, ^15^

Traditional complete denture workflows often involved a series of appointments for medical/dental history, radiographic assessment, psychological assessment, initial impressions, definitive impressions, wax rims/jaw relation records, tooth set‐up try‐in, insertion/clinical remount, and post‐insertion visits with lab workflows co‐ordinated for impression pour ups, custom tray fabrication, wax rim fabrication, wax tooth set‐up, and flasking/processing.^16^ Over time, variations in traditional workflows have emerged, resulting in a diverse number of pathways to produce a set of complete dentures.^17^, ^18^, ^19^, ^20^ With digital technology, an even greater number of pathways are available for providing patients with well‐fitting complete denture prostheses, often with an interweaving combination of analog and digital steps.^15^, ^21^, ^22^, ^23^, ^24^, ^25^, ^26^, ^27^

Despite the potential benefits of incorporating digital technology into complete denture therapy, there are challenges involved in its implementation, and digital techniques have a learning curve.^28^, ^29^ For example, the mobile tissues at the border regions of the denture periphery have been reported to be difficult to accurately capture on intraoral scans; however, clinical techniques have been documented for managing this challenge.^9^, ^29^ Scanning of edentulous ridges can create challenges with the stitching of intraoral images, resulting in an inaccurate scan; although, novel solutions for creating references on the tissue surfaces using materials such as pressure‐indicating paste or flowable composite spheres have been documented.^30^, ^31^ There are challenges with esthetics at the prosthetic gingival margin in CAD‐CAM fabricated complete dentures, which may require further discussion in the literature.^10^, ^32^, ^33^ The convenience of an analog wax rim to record vertical dimension, lip support, centric relation, and wax tooth set‐up try‐ins is challenging to replicate in a digital workflow.^10^ Without an analog try‐in step, a digital preview of a tooth set‐up may be challenging to evaluate without referencing the patient's occlusion and esthetic parameters.^4^ This problem has been partially managed by try‐in “prototype” dentures or try‐in “prototype” prostheses with teeth set in wax.^14^

Patient‐centered outcomes are useful to assess the quality of removable denture therapy.^34^, ^35^, ^36^ According to the systematic review by de Souza et al., patient satisfaction is considered one of the most crucial outcomes in removable prosthodontic clinical trials.^37^ The literature uses different domains to measure patient satisfaction. A patient's level of satisfaction with a set of complete dentures may be related to both clinical and psychological variables.^38^, ^39^ Variables that have been cited to be important to predict patient satisfaction include: personality factors, attitude towards dentures, previous denture experience, quality of the dentures, ridge anatomy, and the patient‐dentist relationship.^40^, ^41^ Cerutti‐Kopplin et al., in a study of 117 complete denture wearers, concluded that chewing ability, maxillary denture stability, mandibular denture retention, and mandibular denture age contributed the most to patient satisfaction.^36^ The Oral Health Impact Profile for Edentulous Patients (OHIP‐EDENT) and the Geriatric Oral Health Assessment Index (GOHAI) are two validated patient satisfaction measurement tools used in complete denture studies.^42^ These two scales can be used to assess patient satisfaction regardless of the method of denture fabrication, whether conventional or digital. With digital technology, there may now be more variables within the process of care that affect patient satisfaction with complete dentures.

The volume of literature on digital denture workflows is increasing; however, there are variations in the degree to which digital technologies are being embraced by clinicians. Clinicians not using digital technology have cited reasons of high initial start‐up costs and lack of perceived advantages over conventional fabrication routes.^43^, ^44^ Articles that provide a compilation of clinical data may be helpful for clinicians seeking an evidence‐based decision on modification of their clinical practice.^45^, ^46^ Particularly, clinicians may be interested in patient‐reported perceptions of digital technologies as well as clinician‐reported outcomes in the field of removable prosthodontics.^47^, ^48^ The volume of literature on traditional complete denture workflows is significant; however, digital dentures, while growing in popularity, still represent an area that is underexplored.

The research gap is particularly notable in directly comparing clinical outcomes and patient perceptions between traditional and digital dentures. This study aims to bridge this knowledge gap by conducting a systematic review following PRISMA guidelines, employing a comprehensive search strategy to evaluate clinician and patient‐reported outcomes associated with both digital and traditional denture workflows. By analyzing these findings, this research seeks to determine whether the digital workflow substantively improves upon conventional methods in terms of clinical efficacy and patient satisfaction.

## METHODS

This systematic review was performed following the Preferred Reporting Items for Systematic Reviews and Meta‐Analyses (PRISMA) guidelines^49^, ^50^, ^51^ using the protocol registered in the International Prospective Register of Systematic Reviews (PROSPERO) with the protocol number CRD42024526069.

The focus question was defined as: “For completely edentulous patients being restored with complete dentures, is there a difference between digital complete dentures and analog complete dentures in terms of patient satisfaction and clinician‐reported outcomes?”

A population, intervention, comparator, and outcomes (PICO) framework was utilized to address the focus question using the following elements: (1) Population: edentulous patients; (2) Intervention: digital complete dentures, digital workflow; (3) Comparator: conventional complete dentures, conventional workflow; (4) Outcomes: patient perceptions of satisfaction and clinician‐reported outcomes.

The following inclusion criteria were applied: articles written in English, articles involving the clinical replacement of missing teeth with removable prostheses, and articles involving digital and analog workflows for removable prosthesis fabrication. Articles were excluded based on the following exclusion criteria: implant‐based therapies, restoration of partially edentulous patients, no comparisons of digital and analog workflows, no reports of patient‐centered data or clinician‐reported outcomes, technique articles, and case reports.

### Search strategy

The search strategy was developed initially by consulting a librarian. An electronic search of the databases with a defined search strategy was completed within PubMed/MEDLINE and Web of Science with a date range from January 2000 to March 2024. A search of the grey literature was completed, and a manual search of article references was performed. The review software Covidence was used for the compilation of data. The search strategy is defined in Tables 1 and 2.

**TABLE 1:** PICO framework and search terms.

Population	“edent*” OR “denture*”
Intervention	“digital*” OR “print*” OR "CAD/CAM" OR “mill*”
Comparator	“conventional” OR “analog” OR “traditional”
Outcomes	“satisfaction” OR “contentment” OR “retention” OR “comfort” OR “appearance” OR “performance” OR “clinician satisfaction” OR “clinician reported outcome” OR “clinician” OR “perception”

**TABLE 2:** Keywords used in each search strategy.

PubMed	Web of Science	Ovid MEDLINE
(((“edent*”[Title/Abstract] OR “denture*”[Title/Abstract]) AND (“digital*”[Title/Abstract] OR “print*” OR“CAD‐CAM”[Title/Abstract] OR “mill*”[Title/Abstract])) AND (“conventional”[Title/Abstract] OR “analog”[Title/Abstract] OR “traditional”[Title/Abstract])) AND (“satisfaction”[Title/Abstract] OR “contentment”[Title/Abstract] OR “retention”[Title/Abstract] OR “comfort”[Title/Abstract] OR “appearance”[Title/Abstract] OR “performance”[Title/Abstract] OR “clinician satisfaction”[Title/Abstract] OR “clinician reported outcome”[Title/Abstract] OR “clinician”[Title/Abstract] OR “perception”[Title/Abstract])	(((TS=(“edent*” OR “denture*”)) AND TS=(“digital*” OR “print*” OR“CAD‐CAM” or “mill*”)) AND TS=(“conventional” OR “analog” OR “traditional”)) AND TS=(“satisfaction” OR “contentment” OR “retention” OR “comfort” OR “appearance” OR “performance” OR “clinician satisfaction” OR “clinician reported outcome” OR “clinician” OR “perception”)	(“edent*” or “denture*”) AND “digital*” OR (“print*” OR“CAD‐CAM” or “mill*”) AND (“conventional” OR “analog” OR “traditional”) AND (“satisfaction” OR “contentment” OR “retention” OR “comfort” OR “appearance” OR “performance” OR “clinician satisfaction” OR “clinician reported outcome” OR “clinician” OR “perception”)

The initial search identified 194 articles from PubMed, 257 articles from Web of Science, and 249 from Ovid MEDLINE. Four additional articles were added from grey literature and handsearching for a total of 704 articles. Three hundred thirty‐seven (337) were identified as duplicates by the Covidence review software and one was manually identified as a duplicate for a total of 338 articles which were removed as duplicates.

### Study selection

Two investigators (AF, DW) independently screened the results of the systematic literature search. Disagreements regarding the title and abstract screening of the studies were resolved through consensus and discussion (K = 0.92). The same two investigators (AF, DW) independently completed a full‐text review of the articles (K = 0.95) and conflicts were resolved by a third and a fourth reviewer (CSD, PM).

**FIGURE 1:**
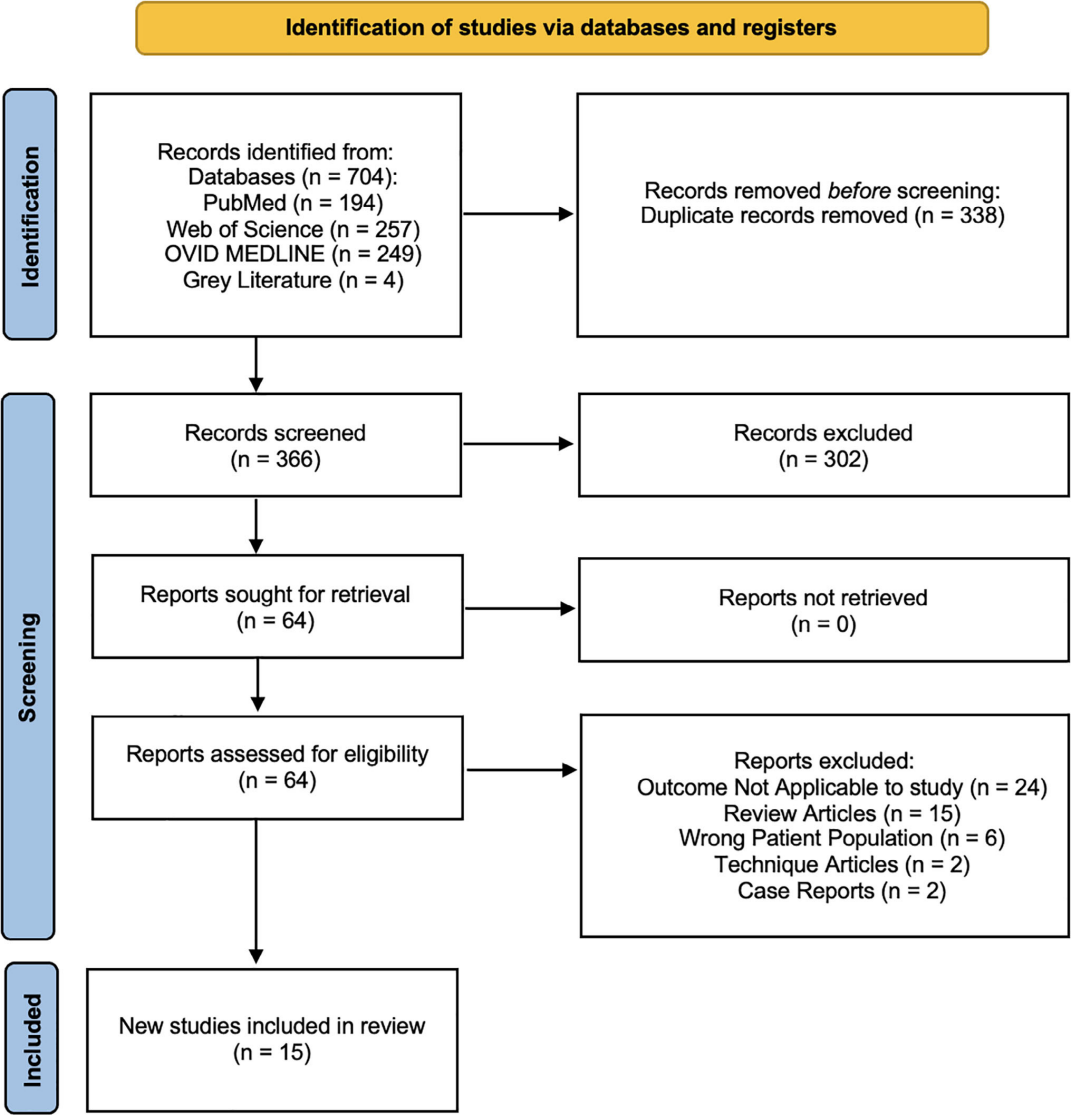
PRISMA flow diagram.

Three hundred sixty‐six (366) articles were screened for title and abstract. Three hundred two (302) articles were found to be irrelevant. Sixty‐four (64) articles were assessed for full‐text eligibility. After assessing the full text of 64 articles, 48 articles were excluded for the following reasons: 24 articles did not report desired outcomes (did not include patient‐centered data or clinician‐reported outcomes), 15 articles were review articles, six articles did not focus on the population of interest (i.e., had partially edentulous patients, or patients with implant‐based therapies), two articles were technique articles, and two articles were case reports. After a full‐text review, 15 articles were included for analysis. The PRISMA flow diagram for the screening process is shown in Figure 1.^52^

### Risk of bias assessment

**FIGURE 2:**
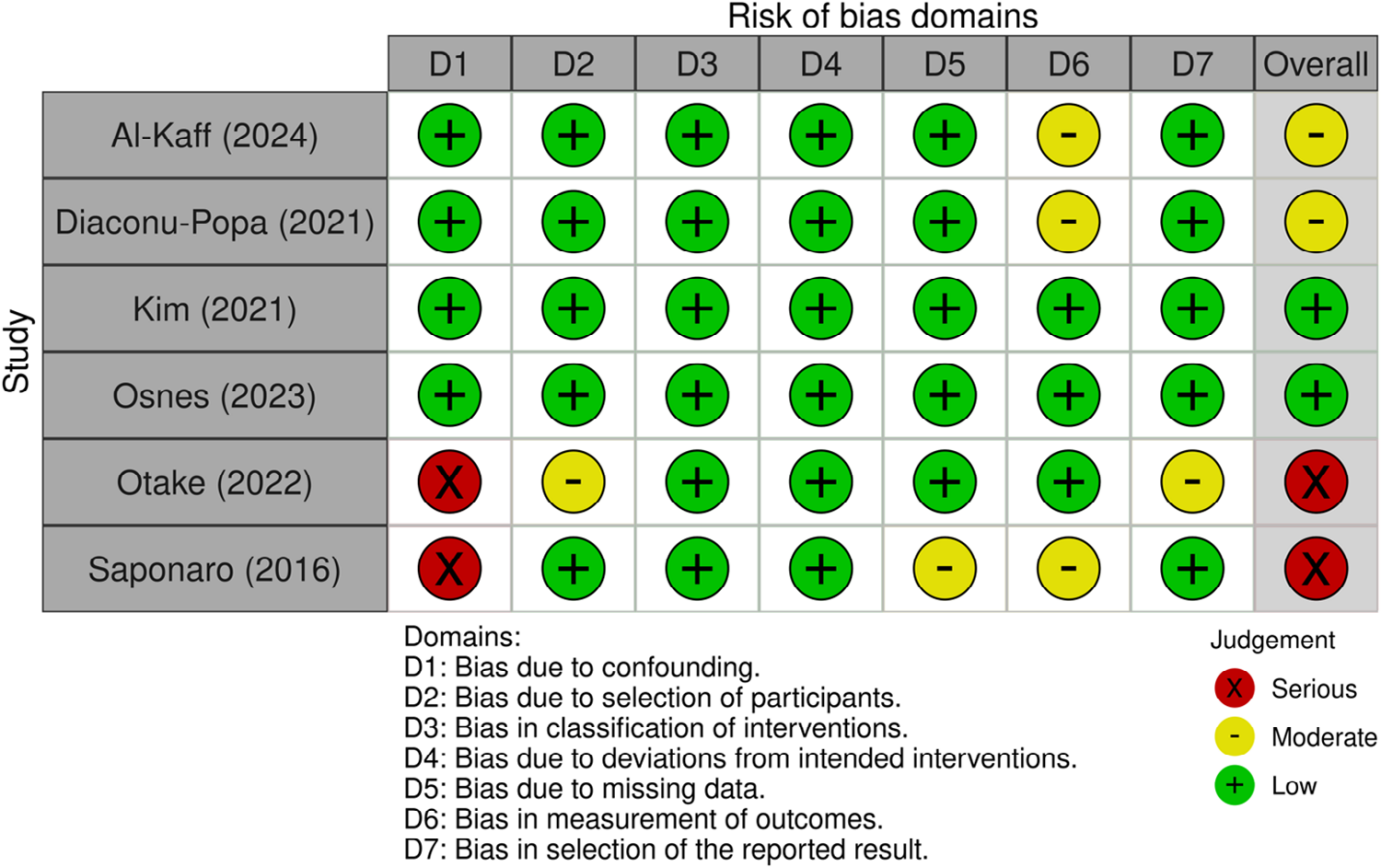
Risk of bias assessment for non‐randomized trials.

**FIGURE 3:**
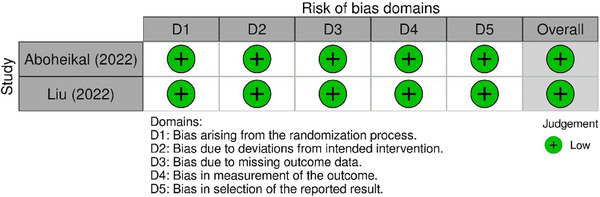
Risk of bias for randomized trials.

**FIGURE 4:**
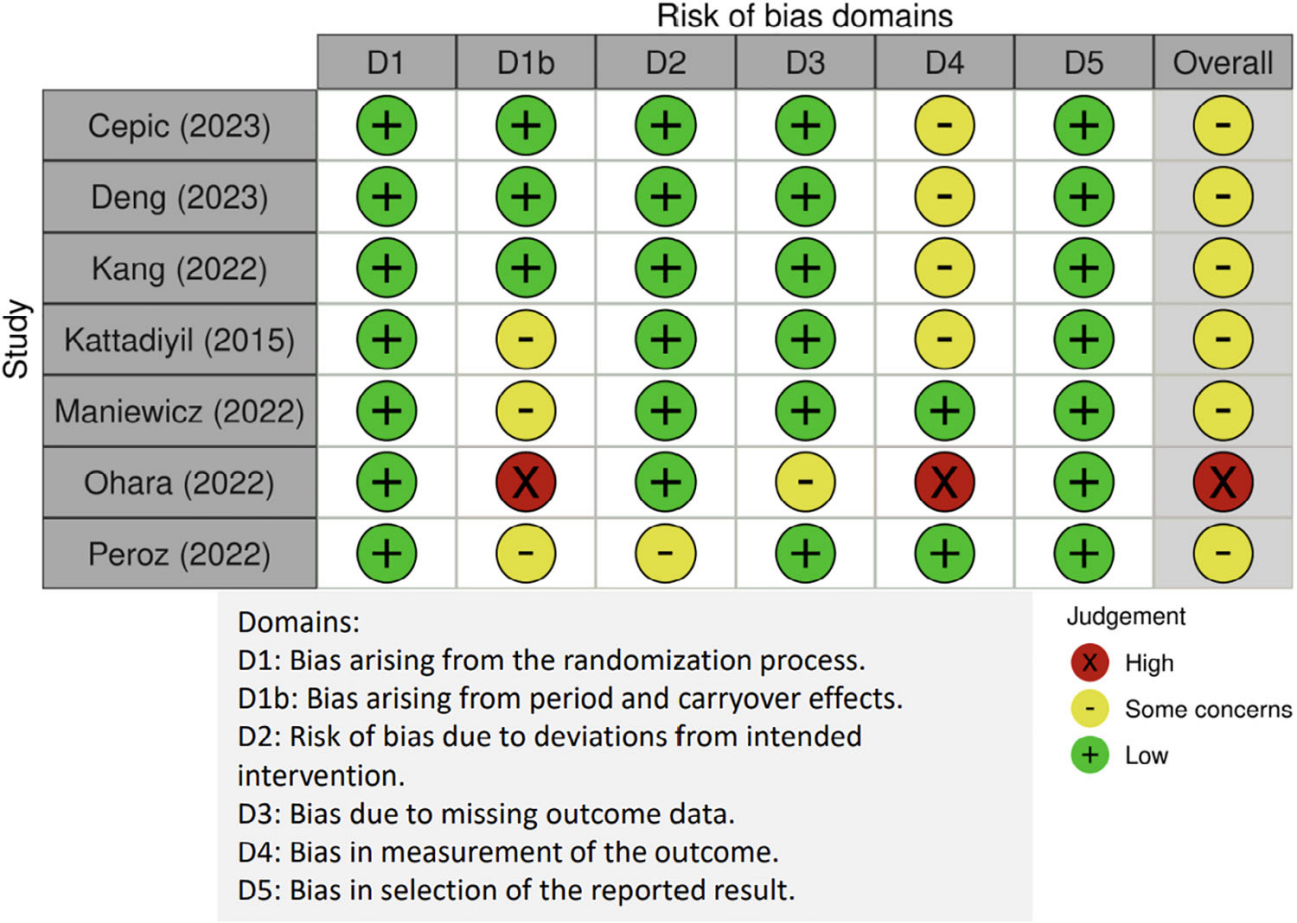
Risk of bias assessment for randomized crossover trials.

One author (JT) completed a risk‐of‐bias assessment using the Cochrane Collaboration risk‐of‐bias tools. Randomized study designs were assessed using either the standard RoB 2 instrument or the RoB 2 instrument for crossover trials, depending on the study design.^53^ Non‐randomized study designs were assessed using the ROBINS‐I instrument.^54^ The results of the assessments were illustrated (Figures 2, 3, 4) using the Cochrane *Robvis* tool.^55^

### Data extraction and analysis

Data extraction was completed by two reviewers (AF, DW), with guidance from the senior author (CSD), using a data extraction table developed by the research team. The details of the data extraction are summarized in Table 3.

**TABLE 3:** Data extraction table.

Author, Year	Country	Study design	Intervention group workflow	Intervention group size	Comparator group workflow	Comparator group size	Follow‐Up	Patient‐reported outcome measures	Patient‐reported outcomes	Clinician‐reported outcome measures	Clinician‐reported outcomes	Conclusions
Aboheikal, 2022^56^	Egypt	Randomized Clinical Trial	Digital scanning of mounted casts, digital design, try‐in, printing or milling for definitive dentures	*n* = 16 patients	Conventional complete denture workflow (limited elaboration on workflow)	*n* = 16 patients	6 months	Patient questionnaire on patient satisfaction items	No significant differences in satisfaction between the groups	Retention force using force gauge	No significant differences in retention force between the groups	Denture fabrication method did not significantly impact patient satisfaction or retention.
Al‐Kaff, 2024^67^	Jordan	Controlled Clinical Trial	Analog preliminary and definitive impressions, wax rims, wax try‐in, intraoral scan or scanning of casts, printed definitive dentures	*n* = 20 patients (same patients in each group)	Analog preliminary and definitive impressions, wax rims, wax try‐in, heat‐processed acrylic definitive denture	*n* = 20 patients (same patients in each group)	4 weeks	OHIP‐EDENT	No statistically significant difference in satisfaction between additively manufactured and conventional denture groups.	Quality assessment with 14‐factor criteria	Additively manufactured dentures were comparable to conventional dentures. Intraoral scanning group had lower overall quality. Teeth arrangement for digital methods was inferior to conventional method.	Patients were satisfied with all denture types. Printed dentures made with intraoral scanning have lower clinical quality than those made cast scanning or conventional methods. Teeth arrangement in both additively manufactured methods was found to be clinically inferior to that of conventional dentures.
Cepic, 2023^58^	Austria	Prospective Randomized Crossover Study	Digital scanning of mounted casts, prototype try‐in with wax, milled definitive dentures	*n* = 10 patients(crossover design)	Analog preliminary and definitive impressions, wax rims, wax try‐in, flasking for definitive denture fabrication	*n* = 10 patients (crossover design)	2 weeks per denture	OHIP‐20	No significant difference in OHIP‐20 scores between groups	Quality of dentures evaluated using Sato score	Higher Sato score for digital dentures for stability. Better polish in conventional dentures.	There was no significant difference in patient satisfaction. Digital dentures had slightly better stability. Conventional dentures had better polish.
Deng, 2023^57^	China	Pilot Study with Crossover Design	Preliminary impressions, cast scanning, prototype try‐in, final impression using prototype, digital design, combination of printing and conventional flasking	*n* = 10 patients (crossover design)	Analog preliminary and definitive impressions, wax rims, wax try‐in, flasking for definitive denture fabrication	*n* = 10 patients (crossover design)	1 week per denture	Visual analog scale scale ranging from 1‐10 on mastication, pronunciation, esthetics, stability, and comfort	No significant differences in patient ratings between the groups	Visual analog scale ranging from 1‐10 on retention, stability, margin extension and occlusal stability. Prosthesis fabrication time and costs.	Occlusal stability of printed dentures was superior to conventional dentures. Costs were less for digital dentures. Prosthesis fabrication time was reduced with digital dentures.	There was no significant difference in patient satisfaction. Digital dentures improved efficiency, provided improved occlusal stability, and reduced costs compared to conventional dentures.
Diaconu‐Popa, 2021^70^	Romania	Comparative Analysis	Analog preliminary and definitive impressions, scanning of definitive casts, digital design, printed or milled definitive dentures	*n* = 10 patients (6 printed dentures and 4 milled dentures)	Analog preliminary and definitive impressions, wax rims, definitive denture fabrication	*n* = 10 patients	1 year	Not reported	Not reported	5‐point quality assessment scale for: surface quality, intraoral adaptation, retention/stability, static/dynamic occlusion, functional restoration	Both digital dentures and conventional dentures were comparable in all categories.	Digital dentures and conventional dentures are comparable in surface quality, adaptation, retention/stability, and static/dynamic occlusion over a 1‐year time period.
Kang, 2022^65^	South Korea	Randomized Crossover Clinical Trial	Intraoral scanning, wax rim fabricated on printed base, digital design, prototype try‐in, printed definitive dentures	*n* = 8 patients (crossover design)	Analog preliminary and definitive impressions, wax rims, wax try‐in, injection moulding for definitive denture fabrication	*n* = 8 patients (crossover design)	1 month per denture	Visual analog scale ranging from 1‐10 on contours, colour, chewing ability, sense of taste, discomfort, retention, and satisfaction	Conventional dentures rated significantly higher on pronunciation and overall satisfaction.	Internal adaptation, masticatory force via pressure‐sensitive film, and masticatory efficiency through the mixing ability index (MAI)	No difference in internal adaptation and masticatory force. Masticatory efficiency was better for conventional dentures.	Conventional dentures were rated higher by patients on pronunciation ability and overall satisfaction. Masticatory efficiency was objectively found to be better for conventional dentures.
Kattadiyil, 2015^62^	United States	Prospective Clinical Study	AvaDent 2‐appointment digital protocol (definitive impressions, interocclusal records and tooth selection at first visit then digital preview and insertion at second visit)	*n* = 15 patients (crossover design)	Analog preliminary and definitive impressions, wax rims, wax try‐in, flasking for definitive denture fabrication	*n* = 15 patients (crossover design)	1 week per denture	Patient questionnaire on retention, appearance, chewing function, comfort, and efficiency	Higher overall patient satisfaction with digital dentures	Checklist used to evaluate for contour, tooth arrangement, fit, esthetics, lip support, centric relation, occlusion, vertical dimension, stability, retention, and phonetics	Faculty evaluations rated digital dentures significantly higher than conventional dentures. Students preferred the digital fabrication pathway.	Patient satisfaction was higher with digital dentures. Objective evaluation found digital dentures to be clinically superior to conventional dentures. Students preferred the digital fabrication pathway.
Kim, 2021^61^	United States	Retrospective Study	Analog preliminary and definitive impressions, wax rims, scanning of mounted casts, digital design, prototype try‐in, printed definitive dentures	*n* = 216 patients	Analog preliminary and definitive impressions, wax rims, wax try‐in, definitive denture fabrication	*n* = 420 patients	1 year	Retrospective chart review for outcomes of: pain/visible lesions, discomfort, lack of retention, lack of stability, esthetic problems, and occlusal problems	Conventional dentures had more pain/visible lesions, and discomfort compared to digital dentures. Digital dentures had more patient esthetic concerns.	Retrospective chart review for outcomes of: number of remakes, number of adjustments, and number of repairs	No differences between digital and conventional dentures regarding remakes, adjustments, or repairs	Patients experienced less overall pain and fewer visible ulcers with printed dentures. Conventional dentures had better esthetic qualities.
Liu, 2022^59^	China	Randomized Controlled Trial	Analog impressions, scanning of impressions, printed casts, wax rims made on printed casts, mounting on articulator, digital design and duplicate denture technique, printed definitive dentures	*n* = 15 patients	Analog preliminary and definitive impressions, wax rims, wax try‐in, flasking for definitive denture fabrication	*n* = 15 patients	6 months	Visual analog scale ranging from 0‐10 on esthetics, ability to speak, ability to chew, stability, and comfort	Digital and conventional dentures were similar for all variables.	Not reported	Not reported	Digital and conventional complete dentures had similar levels of patient satisfaction.
Maniewicz, 2022^33^	Switzerland	Clinical Controlled Crossover Study	Analog preliminary and definitive impressions, scanning of definitive casts, digital design, printed and milled bases for testing	*n* = 19 patients (crossover design)	Analog preliminary and definitive impressions, injection moulded base for testing	*n* = 19 (crossover design)	Not explicitly stated	Visual analog scale on taste, smell, fit, comfort, and smoothness	Digital and conventional bases were the same on all variables except smoothness, which conventional bases were rated higher on	Retention force using force gauge	Digital and conventional bases had similar retention forces	Digital denture bases provide retention and fit similar to those manufactured by conventional methods
Ohara, 2022^63^	Japan	Randomized Crossover Trial	Definitive impressions and interocclusal records with DENTCA trays, digital design and prototype try‐in, printed definitive dentures	*n* = 20 patients (crossover design)	Analog preliminary and definitive impressions, wax rims, wax try‐in, definitive denture fabrication	*n* = 20 patients (crossover design)	Not explicitly stated	OHIP‐EDENT (Japanese Version) and 100‐mm visual analog scale on chewing efficiency, pain, stability, retention, comfort, esthetics, ease of cleaning, phonetics, and satisfaction	Higher satisfaction with conventional dentures for phonetics, ease of cleaning, stability, comfort, and satisfaction.	Number of patient visits, time required for denture fabrication, number of adjustment visits, time required from denture stabilization	Digital dentures required fewer appointments than conventional dentures. There was no difference in the number of adjustment sessions and the time required for denture fabrication.	Digital dentures were inferior for patient satisfaction. Digital dentures required fewer appointments, but the overall treatment time required was similar in both groups.
Osnes, 2023^64^	United Kingdom	Prospective Cohort Study	Analog preliminary and definitive impressions, digital scanning of impressions or casts, wax rims, wax try‐in, printed definitive dentures	*n* = 16 patients (the same patients in each group)	Analog preliminary and definitive impressions, wax rims, wax try‐in, definitive denture fabrication	*n* = 16 patients (the same patients in each group)	Not explicitly stated	Quality of dentures assessed by comfort, stability, and appearance	Patients preferred conventional dentures for comfort, stability, and appearance	Clinician assessment of retention and stability. Clinician preference for jaw relation record (printed or conventional base)	Clinicians preferred printed baseplates. Printed dentures appeared to have reduced retention and stability.	Patients preferred conventional dentures over digital dentures. The workflow for producing printed dentures was not superior to conventional dentures; however, the printed baseplates were preferred by clinicians.
Otake, 2022^69^	Japan	Retrospective Study	Intraoral scanning, putty interocclusal record, digital design, prototype try‐in and definitive impression using prototype, milled definitive dentures	*n* = 20 patients	Conventional complete denture workflow (limited elaboration on workflow)	*n* = 24 patients	Not explicitly stated	100‐mm visual analog scale on general patient satisfaction	Patient satisfaction was higher for the digital denture compared to the conventional denture	Labor costs, total costs, and cost‐effectiveness ratio	Lower labor costs and total costs for digital dentures. Favourable incremental cost‐effectiveness ratio.	Patient satisfaction was higher for digital denture workflow. Digital denture workflow reduces labor costs and total costs.
Peroz, 2022^66^	Germany	Randomized Controlled Crossover Trial	Baltic Denture System 2‐appointment digital protocol with BD keys (analog definitive impressions and interocclusal records, digital design, digitally fabricated denture insertion)	*n* = 16 patients (crossover design)	Analog preliminary and definitive impressions, wax rims, wax try‐in, definitive denture fabrication	*n* = 16 patients (crossover design)	3 months per denture	Oral Health Impact Profile, German Version (OHIP‐G49)	Digital and conventional dentures scored similarly on the OHIP‐G49. Conventional complete dentures resulted in less functional limitation and pain.	Time required for fabrication	Digital dentures required less time to fabricate than conventional dentures.	OHIP‐G49 scores did not differ between conventional and digital dentures. Conventional dentures resulted in less functional limitation and pain. Digital dentures required less time to fabricate.
Saponaro, 2016^60^	United States	Retrospective Survey Study	AvaDent digital denture protocol (details not specified)	*n* = 50 patients who received digital dentures (19 out of 50 participated in the questionnaire)	Not reported	Not reported	Mea*n* = 20 months after insertion of digital dentures	10‐item questionnaire on: chewing ability, smiling ability, speech, ease of cleaning, retention, comfort, overall satisfaction	General satisfaction with digital dentures was high. 70% of respondents found the digital dentures to be “better” than their previous dentures.	Not explicitly stated	Not explicitly stated	Patients had generally positive experiences and outcomes with digital dentures.

## RESULTS

### Patient satisfaction

Most articles found that patient satisfaction did not significantly differ between digital and conventional dentures;^33^, ^56^, ^57^, ^58^, ^59^ however, there were exceptions to this. Some articles found patient satisfaction was improved with digital workflows and others found the opposite to be true. Based on patient responses from a retrospective survey study, Saponaro et al.^60^ found that 70% of patients who were treated at the Ohio State University College of Dentistry Clinics from 2012 to 2014 preferred their new digital dentures over their previous dentures. Respondents in this study rated the digital dentures favourably in terms of chewing ability, smiling ability, speech, ease of cleaning, retention, comfort, and overall satisfaction. In a retrospective chart analysis, Kim et at.^61^ noted that the patients restored with digital complete dentures experienced less pain and denture sores than patients who had conventional complete denture therapy. Kattadiyil et al.^62^ found that patient satisfaction was higher with digital dentures compared to conventional dentures when the AvaDent digital denture protocol was used in a predoctoral dental student setting. Dental students preferred the digital fabrication pathway over the conventional fabrication pathway. In this article, digital denture quality was superior to conventional denture quality based on grades assigned by faculty members using 14‐factor criteria.

Aboheikal et al.^56^ reported that there is no statistically significant difference in patient satisfaction between milled, printed, and conventional dentures and suggested that manufacturing technique is not related to patient satisfaction. Similarly, Liu et al.^59^ found that visual analog scale scores between patients restored with conventional dentures and digital dentures did not show a difference in patient satisfaction under the topics of esthetics, ability to speak, ability to chew, stability, or comfort.

In a randomized crossover trial, Ohara et al.^63^ reported higher satisfaction with conventional dentures and found that only 20% of the patients preferred digital dentures over conventional dentures. In a multi‐centre clinical trial, Osnes et al.^64^ found that there was a general preference for patients to choose conventional dentures over digital dentures.

An overview of the patient satisfaction measurement tools is provided in Table 4 (validated measurement tools) and Table 5 (non‐validated measurement tools).

**TABLE 4:** Studies with validated patient satisfaction measurement tools.

Author	Tools used	Results
Al‐Kaff^67^	The Oral Health Impact Profile for Edentulous Patients (OHIP‐EDENT): The questionnaire uses a 5‐point Likert scale for each time: 0 = “Never,” 1 = “Seldom,” 2 = “Sometimes,” 3 = “Frequently,” 4 = “Always.” Total score: ranges from 0 to 76, with a higher score indicating poorer quality of life. Domains covered by OHIP‐EDENT: Functional Limitation Physical Pain Psychological Discomfort Physical Disability Psychological Disability Social Disability	No statistical difference
Cepic^58^	Oral Health Impact Profile (OHIP‐20): Questionnaire was used to assess the impact of oral health on the quality of life using a 6‐point Likert scale ranging from “never” to “all the time” to measure patient satisfaction. Visual Analog Scales (VAS): 10‐cm scale from “totally dissatisfied” to “completely satisfied” used for specific denture‐related factors including: Ease of cleaning Ability to chew different foods General satisfaction Ability to speak Comfort Esthetics Stability	No significant difference in OHIP‐20 scores
Liu^59^	Patient satisfaction used a 0–10 Visual Analog Scale (VAS) at four different times: Immediately after denture delivery 1 month after delivery 3 months after delivery 6 months after delivery Questionnaire Domains: Esthetics Ability to speak Ability to chew Stability Comfort	Digital and conventional dentures were similar for all variables
Maniewicz^33^	Patient satisfaction was measured using a Visual Analog Scale (VAS) questionnaire to assess different outcomes. The scale ranges from 0 = “least satisfactory” to 10 = “most satisfactory” including: Taste Smell Denture base fit Pain on insertion and removal Smoothness of the denture bases	Digital and conventional bases were the same on all variables except smoothness, which conventional bases were rated higher on
Ohara^63^	Visual Analog Scale (VAS): A 100‐mm VAS was used to quantify different aspects of patient satisfaction, 0 = “least satisfaction,” 100 = “most satisfaction” including: Chewing efficiency Pain Stability Retention Comfort Esthetics Ease of cleaning Phonetics	Higher satisfaction with conventional dentures for phonetics, ease of cleaning, stability, comfort, and satisfaction
Otake^69^	Visual Analog Scale (VAS): 100‐mm VAS with the question “are you satisfied with the current denture?” The scale was anchored with “completely dissatisfied” on one end and with “completely satisfied” on the other end. The assessment was done before and after denture fabrication to evaluate changes in patient satisfaction.	Patient satisfaction was higher for the digital denture compared to the conventional denture
Peroz^66^	Oral Health Impact Profile (OHIP‐49): to evaluate patient satisfaction and the impact of denture type on the quality of life. The OHIP measures patient satisfaction, including: Functional limitations Physical pain Psychological discomfort Physical disability Psychological disability Social disability Handicap	Digital and conventional dentures scored similarly on the OHIP‐G49. Conventional complete dentures resulted in less functional limitation and pain

**TABLE 5:** Studies with non‐validated patient satisfaction measurement tools.

Aboheikal^56^	Patient satisfaction questionnaire assessed five main domains Functional complaints about the dentures Overall masticatory ability Masticating ability for different types of food Effects on mental and daily life Overall denture satisfaction Each domain included several questions: Domains 1 and 2 used scores from 1 to 4 (1 = “never,” 2 = “sometimes,” 3 = “often,” 4 = “always”) Domain 3 used scores from 1 to 3 (1 = “well,” 2 = “moderately,” 3 = “badly”) Domain 4 used scores from 1 to 5 (1 = “never,” 2 = “hardly ever,” 3 = “occasionally,” 4 = “fairly often,” 5 = “very often”)
Deng^57^	Patients scored satisfaction with the dentures on a scale from 0 to 10. The aspects included: Retention Stability Mastication Comfort Esthetics A double blinded manner was implemented as neither the patient nor the evaluating dentist knew which denture was digital and which one was conventional to ensure unbiased evaluation.
Diaconu‐Popa^70^	The study used a scale with the following categories: A: Very good: no negative findings B: Good: 1 negative findings C: Satisfactory: 2 negative findings D: Poor: 3 or more negative findings (clinically satisfactory) E: Clinically unsatisfactory Patients were asked to rate their denture based on this scale and also different aspects were evaluated including: Surface quality Intraoral Adaptation Retention and stability Occlusion
Kang^65^	Patient satisfaction questionnaire included twelve points to assess different aspects of satisfaction with the denture including: Contour of the complete dentures (CDs) Color of denture teeth Chewing ability with dentures Sense of taste with dentures Pronunciation ability when wearing complete dentures Relief of pain or discomfort compared to not wearing them Soft tissue discomfort after placement Overall satisfaction during the first month placement period Retention of the maxillary denture Retention of the mandibular denture Overall score for maxillary CD Overall score for mandibular CD
Kattadiyil^62^	The study used a patient satisfaction questionnaire including: Rating of each denture in terms of retention “stayed in better” Rating of the appearance of each denture Ability to chew and function with the denture Comfort of the denture Efficiency of the denture Preference of which denture the patient chose to wear Each question was rated on a 5‐point Likert scale: 4 = “Excellent” (no negative findings), 3 = “Good” (1 negative finding), 2 = “Fair” (2 negative findings), 1 = “Poor” (3 or more negative findings, but clinically satisfactory), 0 = “Remake” (clinically unsatisfactory)
Kim^61^	The study collected data on complications and maintenance requirements reported by the patient after receiving the dentures.Complications included: Pain and visible ulcers Discomfort Lack of retention Lack of stability Esthetics concerns Occlusal problems
Osnes^64^	Patients provided feedback including: Comfort Stability Appearance of both conventional and 3D‐printed dentures The feedback was collected using a blind assessment, where patients rated their satisfaction with each type of denture. Patients were asked to indicate their preference between conventional dentures and 3D‐printed dentures.
Saponaro^60^	A questionnaire was mailed to patients to assess their satisfaction with CAD‐CAM fabricated complete dentures, patients were given three responses on each statement: “Agree,” “Neutral,” “Disagree.”The questionnaire included the following: Overall satisfaction with the new dentures Improvement in chewing ability Aesthetic appearance of the smile with new dentures Speech improvement or consistency Ease of cleaning compared to old dentures Fit and retention of the dentures Fulfillment of expectations Comfort of the dentures Willingness to recommend the dentures to others Comparison with previous dentures (if applicable)

### Functional outcomes

Kang et al.^65^ noted that patients reported better pronunciation ability for conventionally fabricated dentures over digitally fabricated dentures. This study also found that objectively, using a mixing ability index (MAI) test, conventional complete dentures displayed higher masticatory efficiency. Ohara et al. noted higher satisfaction with conventional dentures regarding phonetics.^63^

### Comfort

In this review, some studies noted that there is comparable satisfaction between digital and conventional dentures in terms of patient comfort. In contrast, other studies highlighted that there is a preference for one approach over another. Kattadiyil et al.^62^ collected patient preferences on digital and conventional dentures and found patient comfort to be improved with a digital workflow. Peroz et al.^66^ used the OHIP‐G49 to compare patient perceptions on digital and conventional complete dentures and found that patients with conventional dentures had less functional limitations and less physical pain than patients with digital complete dentures.

### Esthetics

Al‐Kaff et al.^67^ found that digitally fabricated complete dentures have inferior tooth arrangement esthetics compared to conventional complete dentures; however, this study did not include a subjective patient assessment of esthetics using a similar grading scale to the clinician assessment.

### Clinician‐reported outcomes

Cepic et al.^58^ found that digital dentures offer slightly better stability when compared to conventional dentures using a Sato score assessment.^68^ Kattadiyil et al. stated that digital dentures are more time efficient compared to conventional dentures, and digital workflows are effective in educational settings under faculty supervision. Osnes et al.^64^ stated that although digital technology was preferred in fabricating denture bases to be used during jaw registration, there were challenges regarding tooth positioning and occlusion within the study workflow. Otake et al.^69^ demonstrated that milled dentures were more cost‐effective when compared to conventional dentures. This study illustrated the economic‐related advantages of digital technology. Peroz et al.^66^ found that digital dentures required less fabrication time than conventional dentures.

### Clinical efficiency and time efficiency

**FIGURE 5:**
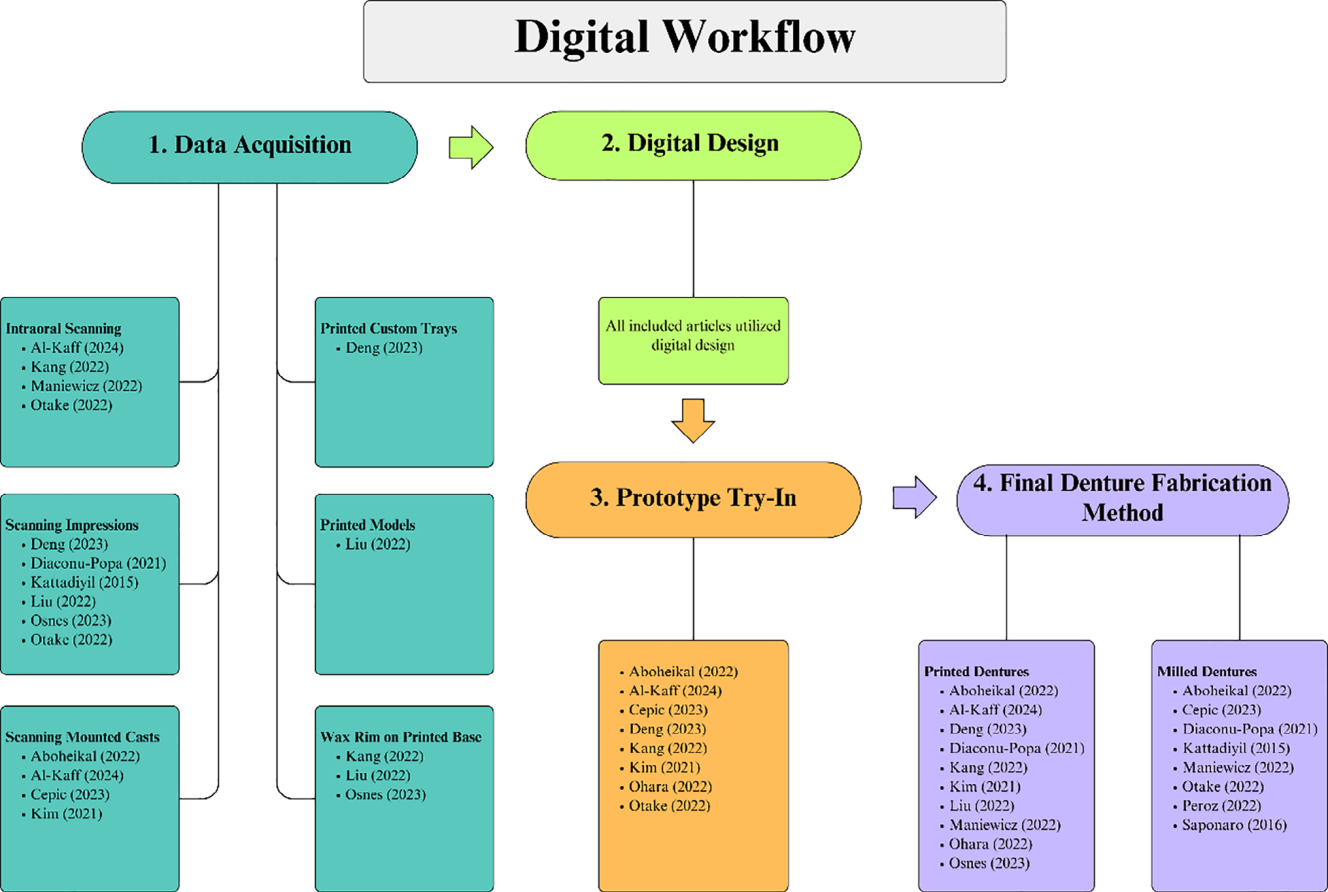
Breakdown of digital workflow steps in each article.

Digital dentures were generally related to time‐saving and improved clinical efficiency. For example, Deng et al.^57^ found that there was significant time‐saving for digital denture fabrication when compared to conventional denture fabrication, and this was attributed to both clinical and laboratory time. Figure 5 illustrates the variations in digital denture fabrication pathways which may result in time‐saving in the process from data acquisition to denture fabrication.

### Denture quality

Within the analyzed articles, the quality of digital dentures was comparable and sometimes better than conventional dentures in specific areas. However, Al‐Kaff et al.^67^ mentioned that digital dentures which use intraoral scanning may have lower clinical quality and retention when compared to conventional dentures or digital dentures which are fabricated from scans of analog casts. Diaconu‐Popa et al.^70^ found that conventional, printed, and milled complete dentures performed equally well in the categories of surface quality, intraoral adaptation, retention/stability, and static/dynamic occlusion during a 1‐year follow‐up period.

### The intersection of patient subjective assessments and clinician objective assessments

Some variables fall under both patient‐reported outcomes and clinician‐reported outcomes. The areas of intersection that the authors were able to identify are denture quality,^64^, ^67^ comfort,^61^ and esthetics.^58^ Denture quality was assessed by both patients and clinicians, often with different metrics. Comfort is another criterion that overlaps between patient and clinician assessments. Patient feedback regarding comfort or fit is subjective and may not align with clinician assessments of fit using instruments such as retention force gauges.^56^ Esthetics was evaluated subjectively by patients and objectively by clinicians based on tooth arrangement, gingival contour, and using knowledge of pink and white esthetic scores. Reports of patient satisfaction were not always supported by positive clinician‐reported outcomes.^33^, ^56^, ^62^

## DISCUSSION

This systematic review compared patient satisfaction and clinician‐reported outcomes for complete dentures fabricated with digital and conventional workflows. In general, there was little difference in patient satisfaction levels between digital and conventional complete dentures across several studies and this may indicate that the mode of fabrication (digital versus conventional) did not directly influence patient satisfaction. This is supported by existing literature^25^, ^71^, ^72^, ^73^ which suggests that while digital dentures can offer certain advantages such as reduced fabrication time and improved fit, these technical benefits do not necessarily translate to higher patient satisfaction. However, evidence suggests that there is a preference for milled dentures over conventional dentures and printed dentures. Further investigation into this aspect is required.

Studies in the literature have shown that the patients reported greater satisfaction with digitally fabricated complete dentures. For example, Mubaraki et al.^25^ stated in their review that patients preferred digital dentures due to their better fit and reduced chair time as well as the fewer post‐insertion visits. However, our study reported limitations with the digital workflow especially with establishing occlusion and with digital tooth setups. This result is consistent with the narrative review written by Anadioti et al.^74^ about printed removable complete dental prostheses which stated that the limitation of this technology is poor esthetics, retention, inability to balance the occlusion, as well as poor printing resolution. It is important to note that only printed dentures were included in their review.

The studies in this review reported that denture retention was generally satisfactory with digital dentures. This aligns with the systematic review completed by Chocano et al.^75^ which stated that digitally fabricated complete dentures had better retention and better adaptation to the tissues compared to conventional complete dentures.

It was concluded in our review that digital dentures required fewer appointments and less clinic time as reported by Deng et al.^57^ and this is consistent with the systematic review that was done by Schweiger et al.^71^ which stated that the use of digital technology in the production of a complete denture may reduce the number of appointments needed in the dental office. As the steps prior to digital design of complete dentures are not routinely completed digitally, it is clear that research is needed to support development of a predictable protocol for digital capture of the functional borders for complete dentures.

A range of studies have reported on the clinical outcome and clinician‐reported outcomes of digitally fabricated dentures. Anadioti et al.^74^ and Wagner et al.^14^ highlighted that 3D printing and additive manufacturing can improve clinical workflow and reduce clinic costs. This is consistent with the findings in our systematic review. Jafarpour et al.^72^ and Smith et al.^73^ further supported the previous findings noting that the time and cost savings with digital dentures may be significant.

It is important to emphasize that there was little consensus as to the optimal digital workflow steps used in the studies, meaning there was no specific protocol for implementing digital dentistry in complete denture fabrication. Some studies had only the final denture fabricated digitally while others had multiple steps such as intraoral scanning. For this reason, it was difficult to compare the studies without determining the extent of the digital workflow utilized. This heterogeneity of digital workflow presents a challenge in drawing a definitive conclusion. Future studies might aim to standardize digital workflow protocols for a more predictable comparison and data analysis.

Some limitations of this systematic review are the small sample sizes and the short follow‐up periods of the included studies. This might be due to the novelty of the digital denture fabrication technology. These factors necessitate further research with larger cohorts and longer follow‐up periods. It is also important to conduct studies with standardized digital workflow protocols to fully understand the implications of the digital workflow versus the conventional denture fabrication workflow.

Establishing effective workflow patterns within digital denture technologies may require a background in conventional denture treatment. Traditional removable prosthodontic workflows commonly involve analog wax rims to establish appropriate vertical and horizontal ridge relationships.^16^ Obtaining useful records using wax rims requires significant clinical skill and judgment to become efficient, accurate, and consistent in clinical technique. If a wax rim is not adjusted and utilized correctly to provide accurate clinical information to the laboratory, the resulting tooth set‐up may require significant modification.^76^ The concepts involved in establishing an appropriate wax tooth set‐up within physiologic boundaries may best be described as a combination of art and science based on classical prosthodontic literature.^77^, ^78^, ^79^ The challenges encountered in fabricating and assessing analog wax tooth set‐ups may be similar to those encountered with CAD‐CAM digital tooth set‐ups.^28^, ^62^ A significant benefit to the use of an analog wax rim or wax tooth set‐up is the ability to add and subtract from the rim while clinically evaluating the patient. In a digital context, this ability to add and subtract still exists; however, the ability to try in the set‐up requires either a printed prototype or a unique solution such as a Wagner Try‐In (WTI) prototype.^14^ In conventional workflows, an adequate number of appointments exists to verify jaw relation records before proceeding to finalize the prosthesis. In CAD‐CAM denture protocols with a reduced number of appointments, the jaw relation record verification stage may be absent, which may or may not result in complications during a digital workflow.^4^ Sun et al. cautioned clinicians of the challenges in completing a digital tooth set‐up without a wax rim which provides data for the fullness of the upper lip and esthetic parameters.^10^ A knowledge of relationships between natural tooth positions and dimensions to intraoral or extraoral anatomical landmarks is as helpful in a digital workflow as it is in a conventional workflow in the laboratory.^80^, ^81^, ^82^, ^83^, ^84^ It may be such that merging of a facial scan or other digital data with digital casts will become a more commonly used procedure in the future for overcoming some limitations of a digital tooth set‐up and further research in this field is warranted.^85^, ^86^

One of the goals of this review was to determine if there is a difference in patient‐reported outcomes based on whether a set of complete dentures was fabricated digitally or conventionally. There may be many confounding factors regarding patient‐reported outcomes with complete dentures which cannot be easily determined by reading the literature alone; therefore, limiting the value of the conclusions of our article.^87^ Although well‐fabricated prostheses will contribute to patient satisfaction, it has been reported that other factors related to patient adaptability and self‐perception may also play a role in overall satisfaction with complete denture therapy.^41^, ^88^ In addition, one cannot underestimate the importance of the interaction between the clinician and the patient during the fabrication process of complete dentures, both in analog and digital workflows, where the unique psychological aspects and mental attitudes of a patient can be assessed and managed, to help guide patient expectations and improve satisfaction with treatment.^89^, ^90^, ^91^, ^92^

It was difficult to conclude whether a digitally fabricated set of complete dentures consistently resulted in a higher level of patient satisfaction than a conventionally fabricated set of complete dentures. This was partly due to the inconsistency between articles on patient satisfaction measurement tools as well as differences in classification of a “fully digitally fabricated” prosthesis. The authors feel that validated scales, such as the OHIP‐EDENT^93^ may be beneficial to allow for comparison between studies. In addition, within this review, the authors found that the most common visual analog scale^94^ topics were: speech, comfort, esthetics, stability, and chewing efficacy. Future studies may consider using these topics for visual analog scales to allow for comparisons to studies already completed in this field. As further data is collected and compared to the studies included in this review, the advantages of digitally fabricated complete dentures may become clearer.

Future studies may need to consider the most optimal methods for designing a washout period. There may be ethical and logistical challenges with forcing patients to be without their dentures for any period of time during the washout period between the trial period with a digital denture and the trial period with a conventional denture; however, a washout period is important to ensure a high‐quality methodology is in place. If a parallel arm trial is conducted with one group receiving digitally fabricated complete dentures and another receiving conventionally fabricated complete dentures, it may be advisable to match the participants based on clinical or psychological variables so that confounding variables are minimized; however, there are logistical and ethical challenges to this as well. Finally, future studies should consider the length of follow‐up that may be required not only to assess patient's initial satisfaction with removable prostheses, but also their satisfaction in the months and years ahead. Maintenance needs of digitally fabricated (printed or milled) prostheses may be an area that requires further investigation.

Removable complete dentures have inherent limitations, and patient satisfaction with this prosthesis design may be linked to the patient's psychological makeup and the communication abilities of the treating clinician.^95^ It may be such that emotionally understanding the effects of tooth loss and the challenges of the edentulous state may be important to patient management.^90^, ^96^, ^97^ “Meeting the mind of the patient before meeting the mouth of the patient”^98^ may be equally as applicable in digital denture workflows as it is in conventional denture workflows, although further research is warranted in this field. A patient's mental classification^99^ may be a useful baseline variable to record for each patient involved in future research in the field of digital and conventional dentures, although there may be methodological challenges to this. The differences between digital and conventional complete denture fabrication methods may play less of a role than the interpersonal skills of the clinician, provided the clinician has a general understanding of the concepts of complete denture therapy and is working with a patient of sound mind.

Although this article has attempted to seek out differences in outcomes for digitally fabricated versus conventionally fabricated complete dentures, in truth, most articles have “hybrid” workflows where the digital and analog pathways merge and interweave themselves on the path to the final prosthesis.^100^, ^101^ Although a fully digital workflow is not a definition found in the Glossary of Prosthodontic Terms (GPT‐9), a true digital workflow could be defined as one in which no physical casts are generated, and all treatment is completed in a virtual environment. For this to occur, the impression would be made through a digital scan, along with a digital articulation, digital tooth set‐up, and milled or printed fabrication pathway. Articles within this review generally followed a digital workflow which involved making analog impressions, followed by a digital workflow either with or without a try‐in prototype. Many of the original digital denture systems in the literature, including AvaDent and DENTCA, involve an analog impression stage and analog interocclusal records involving a centric relation record using a modified form of traditional gothic arch tracing.^15^, ^21^ Although digital dentistry has introduced methods that expand the horizons of dental care,^2^, ^102^ it appears that many concepts and techniques from classical prosthodontics still have a place in modified workflows. A clinician will benefit from an understanding of conventional denture workflows (impression making, interocclusal records, etc.) before attempting to proceed with a digital system.

Case studies were not included in the final analysis since these studies had a low level of evidence. However, these studies^21^, ^101^ provide significant background information on digital complete denture therapy in a manner that is simpler to understand through illustrations and photographs than the studies that are technically higher on the evidence pyramid. Jurado et al.^101^ discussed the value of a combination of analog and digital techniques in managing severely resorbed ridges. Pereyra et al.^21^ discussed the techniques and concepts involved in the DENTCA digital denture system and compared the system to conventional complete denture fabrication pathways. A reader who is interested in beginning to experiment with digital complete denture therapy could benefit from reading these articles in addition to understanding the broader conclusions from higher levels of evidence.

Articles within this review ranged in their degree of perceived bias. Bias has been found to be a significant challenge to overcome in prosthodontic research.^103^ The risk of bias assessment was challenging for some articles, given some limitations of reported data within the articles. The authors are aware that excluded information may have been solely related to word count limits, and it appears that all articles attempted to limit bias to the degree possible. There are always inherent challenges in evaluating and critiquing the reports of research studies.^104^ All guidelines for the Cochrane Collaboration risk‐of‐bias tools were followed including the algorithms to arrive at the ranking of each article for risk of bias.

A prudent clinician who is deciding to implement a digital denture workflow may first turn to the scientific literature and look for an evidence‐based rationale.^105^, ^106^, ^107^ Digital workflows have been shown to be more efficient; however, they have also presented new ethical challenges to the profession.^108^ It may be such that clinicians/researchers/academic institutions implementing digital workflows for the first time and comparing them to conventional workflows may encounter significant barriers to their implementation.^108^

Patients tended to prefer conventional dentures over printed digital dentures,^32^, ^63^, ^64^, ^67^ whereas they tended to prefer digital dentures when the prosthesis was milled.^58^, ^62^, ^69^ This suggests that the method of digital denture fabrication employed might influence patients’ preferences.

The authors hypothesize that first‐time users may feel ethically inclined to ensure digital workflows deliver equivalent results to conventional “historical gold standard” methods, although more research is required to investigate this hypothesis. A future potential ethical concern in the field of CAD‐CAM denture research is the influence of market pressures and conflicts of interest on research design and reporting. Given the rapidly advancing nature of digital technology, CAD‐CAM research may be at risk for industry bias or conflicts of interest due to the influence of market competition in trying to demonstrate the superiority of one technology or method over another.^109^, ^110^ Clinicians and researchers should remain vigilant to this possibility to ensure all research is completed without commercial influence and with the best interests of patient care in mind.

## CONCLUSIONS

Patient satisfaction and clinician‐reported outcomes were measured differently among the 15 studies in this systematic review. Although digital dentures provided favorable outcomes like conventional dentures, overall superiority or comparability of these treatment options remains unclear and requires further research. It is essential to differentiate between milled and printed dentures, given their dissimilar outcomes. Milled digital dentures generally showed better patient acceptance. In contrast, printed dentures faced challenges in areas such as quality and esthetics, impacting patient preference. Function, esthetics, and comfort are components of patient satisfaction that clinicians strive to provide during patient care. It remains a challenge to standardize measurement of these outcomes while minimizing the burden to patients and clinicians involved in clinical trials. Clinicians choosing to adopt digital technology into traditional removable prosthodontic workflows will need to manage the learning curve and include strategies to support positive outcomes for patient satisfaction. Along with efforts to develop a fully digital workflow for complete dentures, future research on the impact of digital workflows on complete denture outcomes requires more standardized research methods to enable comparisons to be made in a meta‐analysis.

## CONFLICT OF INTEREST STATEMENT

There are no conflicts of interest to declare for any of the authors.

## FUNDING INFORMATION

No financial support was received for this research.
